# Survival Outcomes in Transthyretin Cardiac Amyloidosis in the Tafamidis Era: Real-World Evidence from Greece

**DOI:** 10.3390/diagnostics16203373

**Published:** 2026-10-16

**Authors:** Freideriki Eleni Kourti, Alexios S. Antonopoulos, Vasileios Kytinos, Theodoros Tsampras, Alexandrina Kouroutzoglou, Dimitrios Kyriakopoulos, Maria Eleni Koilakou, Pinelopi Karampina, Vasiliki Gardikioti, Dimitrios Terentes-Printzios, Alexandros Kasiakogias, Ioannis Leontsinis, Christina Chrysohoou, Maria Koutelou, Konstantinos Toutouzas, Konstantinos Tsioufis, Charalambos Vlachopoulos

**Affiliations:** 1 Cardiogenetics Unit, National Cardiac Amyloidosis Centre, 1st Cardiology Department, Hippokration Hospital, National and Kapodistrian University of Athens, 11527 Athens, Greece; 2 Department of Nuclear Medicine, Kosmoiatriki, 11254 Athens, Greece; 3 Department of Nuclear Medicine, Bioiatriki, 11526 Athens, Greece

**Keywords:** amyloidosis, transthyretin amyloid cardiomyopathy, survival, tafamidis

## Abstract

**Background:** Diagnoses of transthyretin amyloid cardiomyopathy (ATTR-CM) have increased in recent years, with a growing proportion of patients being identified at earlier disease stages. However, temporal trends in ATTR-CM diagnosis and survival during the tafamidis era in the Greek population remain largely unknown. **Methods:** This retrospective study included 75 patients with ATTR-CM treated with tafamidis. Baseline demographic, clinical, laboratory, and echocardiographic characteristics were assessed, and survival outcomes were subsequently evaluated. **Results:** Among 75 tafamidis-treated patients, 14 deaths (19%) occurred during a median follow-up of 20 months [IQR 10.0–30.5] with an estimated 30-month survival of 75%. Kaplan–Meier analysis demonstrated significantly poorer survival among patients aged ≥80 years (log-rank *p* = 0.013), those with NYHA class III–IV (log-rank *p* = 0.018), and across progressively more advanced NAC stages I–III (global log-rank *p* < 0.001). In contrast, survival did not differ significantly according to LVEF <50% versus ≥50% (log-rank *p* = 0.863) or maximal wall thickness ≥15 mm versus <15 mm (log-rank *p* = 0.801). After Holm correction across five comparisons, only NAC stage remained statistically significant (adjusted *p* < 0.001). **Conclusions:** In this real-world Greek cohort of patients with ATTR-CM treated with tafamidis, the estimated 30-month all-cause mortality was 25% and more advanced NAC stage was associated with poorer survival.

## 1. Introduction

Transthyretin amyloid cardiomyopathy (ATTR-CM) is a myocardial disease characterized by the extracellular deposition of amyloid fibrils, leading to progressive heart failure with a poor prognosis and arrhythmias [1,2]. Despite being previously considered a rare entity, over the last decade ATTR-CM has gained recognition as an important cause of heart failure with preserved ejection fraction (HFpEF), or as a concomitant condition in specific groups of patients, such as those with aortic stenosis or carpal tunnel syndrome [3,4,5,6,7].

The integration of non-invasive methods, particularly 99mTc-DPD/PYP/HMDP bone scintigraphy and cardiac magnetic resonance (CMR) findings, in the diagnostic algorithm has substantially improved disease recognition and enabled earlier diagnosis, ultimately translating into better patient outcomes [1,8,9]. Data from the UK National Amyloidosis Centre have demonstrated an increase in referrals of patients with suspected ATTR-CM after bone scintigraphy and CMR between 2002–2021, accompanied by a significant increase in wild-type ATTR-CM diagnoses (2017–2021: 66% versus 2007–2011: 54%, *p* < 0.001) [10]. Despite the increasing number of referrals over time, only a proportion of patients are referred at an early stage of disease, highlighting the persistent challenge of timely diagnosis [11].

Tafamidis, a stabilizer of the TTR tetramer, became the first FDA-approved targeted disease-modifying therapy for ATTR-CM in 2019. The ATTR-ACT trial (Tafamidis Treatment for Patients with Transthyretin Amyloid Cardiomyopathy) demonstrated its safety and efficacy in patients with ATTR-CM, significantly reducing all-cause mortality and cardiovascular-related hospitalizations, while attenuating the worsening of functional capacity and quality of life [12]. Since then, novel therapies, including acoramidis and vutrisiran, have shown clinical benefit in large randomized clinical trials and have expanded the therapeutic landscape of ATTR-CM [13,14]. A meta-analysis integrating real-world data showed that ATTR-targeted treatments reduced mortality by 39% (risk ratio [RR] 0.61, 95% confidence intervals [CIs]: 0.52–0.70). A sensitivity subgroup analysis for randomized clinical trials only confirmed a 28% reduction in all-cause mortality (RR 0.72, 95% CI: 0.60–0.86) [15].

Furthermore, a meta-analysis of the natural history of ATTR-CM, conducted before the widespread availability of newer disease-modifying therapies, reported 1-, 2-, and 5-year survival rates of 91.9%, 76.0%, and 48.0%, respectively, among patients with wild-type ATTR-CM. Patients receiving tafamidis or patisiran demonstrated significantly higher survival than those managed with standard heart failure therapy alone, with 2-year survival rates of 79.9% versus 72.4% and 5-year survival rates of 64.0% versus 45.8%, respectively (*p* < 0.05), highlighting that disease-modifying therapies have significantly altered the natural history of ATTR-CM [2].

Patients enrolled in clinical trials must meet specific inclusion and exclusion criteria, which may limit the generalizability of their findings to broader patient populations represented in real-world studies and registries. Additionally, real-world cohorts may reflect differences in healthcare systems, reimbursement policies, and access to treatment, factors that are underrepresented in randomized clinical trials.

Therefore, we aimed to characterize temporal trends in disease stage at diagnosis and clinical outcomes among patients with ATTR-CM referred to a tertiary amyloidosis centre in Greece during the tafamidis era.

## 2. Methods

### 2.1. Study Design

This was a retrospective, single-centre observational cohort study including consecutive patients with ATTR-CM referred to the Cardiogenetics Unit of the 1st University Cardiology Clinic, Hippokration General Hospital, Athens, Greece, between January 2019 and March 2025 for evaluation of suspected cardiac amyloidosis. Patients were identified through institutional registries and electronic medical records. All consecutive adult patients with a confirmed diagnosis of ATTR-CM established according to contemporary international diagnostic criteria were eligible for inclusion [8]. The study aimed to describe the clinical phenotype at presentation, explore disease stage across diagnostic periods, and evaluate survival among patients treated with tafamidis. The study was reported in accordance with the Strengthening the Reporting of Observational Studies in Epidemiology (STROBE) guidelines (Supplementary File S1).

ATTR-CM was classified into wild-type ATTR (ATTRwt) or hereditary ATTR (ATTRv) according to genetic testing results. Genetic testing was systematically offered to all patients; however, some patients did not proceed with testing. Patients without available genetic testing results were therefore classified as “not subtyped” and were not presumed to have ATTRwt. Patients with evidence of immunoglobulin light-chain (AL) amyloidosis or other forms of cardiac amyloidosis were excluded. Patients with insufficient clinical information at baseline, missing essential diagnostic investigations, or unavailable follow-up data were also excluded from the final analyses.

The study was conducted in accordance with the ethical principles outlined in the Declaration of Helsinki and complied with local regulatory requirements. Institutional Research Ethics Committee approval was obtained before data collection.

### 2.2. Confirmation of ATTR-CM Diagnosis

All patients underwent a standardized diagnostic work-up performed as part of routine clinical practice. Initial evaluation included a detailed medical history, physical examination, twelve-lead electrocardiography, laboratory testing, transthoracic echocardiography, and multimodality cardiac imaging when clinically indicated.

Patients were screened for ATTR-CM when unexplained left ventricular wall thickening (≥12 mm) was present together with one or more clinical, electrocardiographic, imaging, or extracardiac “red flags” suggestive of systemic transthyretin amyloidosis. Clinical suspicion was based on combinations of characteristic findings, including heart failure with preserved or mildly reduced ejection fraction, low-flow low-gradient aortic stenosis, disproportionate left ventricular hypertrophy relative to the degree of hypertension, unexplained elevation of cardiac biomarkers, atrial fibrillation, conduction disease requiring pacemaker implantation, intolerance to conventional heart failure therapies, bilateral carpal tunnel syndrome, lumbar spinal stenosis, spontaneous distal biceps tendon rupture, peripheral or autonomic neuropathy, orthostatic hypotension, and previous orthopaedic procedures including hip or knee arthroplasty.

The diagnosis of ATTR-CM followed contemporary international recommendations. Non-invasive diagnosis was established in patients demonstrating grade 2 or grade 3 myocardial tracer uptake on technetium-labelled bone scintigraphy (99mTc-DPD or 99mTc-PYP), in the absence of evidence of monoclonal immunoglobulin production. All patients therefore underwent serum free light-chain quantification, along with serum and urine immunofixation electrophoresis to exclude AL amyloidosis. In cases with equivocal scintigraphy findings, atypical clinical presentation, coexistence of monoclonal gammopathy, or diagnostic uncertainty, histological confirmation was obtained by endomyocardial biopsy or extracardiac tissue biopsy with Congo red staining and amyloid typing using immunohistochemistry or mass spectrometry, according to local practice.

Patients with hereditary disease underwent sequencing of the *TTR* gene for identification of pathogenic variants. The date of diagnosis was defined as the date on which ATTR-CM was first confirmed according to contemporary diagnostic criteria.

### 2.3. Data Collection

Baseline variables corresponded to the patient’s clinical status at the time of ATTR-CM diagnosis. Demographic variables included age at diagnosis, sex, and calendar year of diagnosis. Disease subtype was classified as ATTRwt or ATTRv, or not subtyped. Clinical characteristics included New York Heart Association (NYHA) functional class, history of HFpEF, chronic kidney disease (CKD), and prior implantation of a pacemaker or an implantable cardioverter-defibrillator. HFpEF was defined as documented clinical heart failure in the presence of a left ventricular ejection fraction (LVEF) ≥50%. Left ventricular hypertrophy (LVH) was defined as a maximal wall thickness ≥12 mm on transthoracic echocardiography. Prior aortic valve interventions in patients with severe aortic stenosis performed before tafamidis initiation were recorded, including transcatheter and surgical aortic valve replacement. ATTR-associated extracardiac manifestations were systematically recorded. Laboratory assessment included measurements obtained closest to the date of diagnosis. Cardiac biomarkers included high-sensitivity cardiac troponin I (hs-cTnI) and N-terminal pro-B-type natriuretic peptide (NT-proBNP). Hs-cTnI was measured using the Alinity i STAT High Sensitive Troponin-I assay (Abbott, Longford, Ireland), a two-step chemiluminescent microparticle immunoassay, performed on the fully automated Alinity i analyzer. Elevated hs-cTnI was defined as a value above the sex-specific 99th-percentile upper reference limit (>34.2 pg/mL in men and >15.6 pg/mL in women). Renal function was evaluated using serum creatinine and estimated glomerular filtration rate (eGFR) calculated using the CKD-EPI equation. When multiple measurements were available within the diagnostic period, values closest to the diagnostic evaluation were used for analysis.

### 2.4. Echocardiographic Assessment

Comprehensive transthoracic echocardiography was performed using commercially available ultrasound systems by experienced echocardiographers according to contemporary recommendations from the American Society of Echocardiography and the European Association of Cardiovascular Imaging. Echocardiographic assessment included measurement of LVEF, using the biplane Simpson method whenever image quality permitted, and left ventricular maximal wall thickness (MWT). Global longitudinal strain was recorded when available but was not included in the primary statistical analyses because of incomplete availability throughout the study period. For survival analyses, LVEF and MWT were selected as the principal echocardiographic variables because of their established prognostic significance and consistent availability.

### 2.5. Disease Staging

Disease severity at diagnosis was assessed using the National Amyloidosis Centre (NAC) staging system, which stratifies patients according to NT-proBNP concentration and renal function. Patients were classified into stages I, II, or III according to established biomarker thresholds using NT-proBNP and estimated glomerular filtration rate (eGFR). NYHA functional class was additionally recorded as a complementary measure of functional impairment and symptomatic disease burden. Both classification systems were evaluated descriptively to characterize disease severity at presentation and to explore differences in disease stage at diagnosis during the study period.

### 2.6. Follow-Up and Outcomes

Survival analyses included patients who initiated tafamidis free acid at 61 mg once daily following diagnosis. The median interval between ATTR-CM diagnosis and tafamidis initiation was 3 weeks [IQR 1–7]. The index date for survival analyses was defined as the date of treatment initiation. Patients were followed longitudinally through dedicated outpatient clinics, hospital electronic records, and linkage with the national healthcare system whenever available. Mortality status was confirmed through review of hospital records, outpatient documentation, and official national death registries when accessible. The primary outcome was all-cause mortality. Follow-up time was calculated from tafamidis initiation until death or the date of last available clinical follow-up for censored patients. Patients who switched treatment (*n* = 3) were right-censored at the date of treatment switch. No patient permanently discontinued tafamidis during follow-up. Patients who remained alive at the end of the observation period were censored at their last documented clinical contact.

### 2.7. Statistical Analysis

Continuous variables were initially evaluated for normality using visual inspection of histograms and the Shapiro–Wilk test. Normally distributed variables are presented as mean ± standard deviation (SD), whereas skewed variables are summarized as median and interquartile range (IQR). Categorical variables are presented as absolute frequencies and percentages. Baseline characteristics were compared using Student’s *t*-test or the Mann–Whitney *U* test for continuous variables, as appropriate, and Pearson’s χ^2^ test or Fisher’s exact test for categorical variables. NAC stage distributions were described graphically for the two diagnostic periods. For period-based comparisons, patients were classified according to year of diagnosis into two predefined periods: 2019–2021 (January 2019 through December 2021) and 2022–2025 (January 2022 through March 2025). Differences in NAC stage distribution between the two diagnostic periods were assessed using Fisher’s exact test because of small expected cell counts. To assess temporal changes in disease stage, ordinal logistic regression was performed with NAC stage (I, II, and III) as the dependent variable and calendar year of diagnosis as a continuous predictor. The proportional odds assumption was assessed using the test of parallel lines. Results are reported as odds ratios (ORs) per one-year increase in calendar year with 95% CIs.

Overall survival was estimated using the Kaplan–Meier method. Kaplan–Meier survival estimates were presented with 95% pointwise confidence intervals calculated using Greenwood’s variance and a log-minus-log transformation. Survival curves were compared using the log-rank test. Prespecified subgroup analyses were performed according to age (<80 versus ≥80 years), NAC stage (I, II, III), NYHA functional class (I–II versus III–IV), LVEF (≥50% versus <50%), and MWT (≥15 mm versus <15 mm). To account for multiple comparisons across the five log-rank analyses, *p*-values were adjusted using the Holm method.

Cox proportional hazards regression was used to estimate hazard ratios (HRs) and 95% CIs for all-cause mortality. Univariable Cox proportional hazards models were performed for age ≥80 years, NYHA functional classes III–IV, and NAC stage treated as an ordinal variable (per one-stage increase), LVEF ≥50% versus <50%, and MWT ≥15 mm versus <15 mm. Because no deaths occurred among patients aged <80 years, the univariable age comparison was analysed using Firth-penalized Cox regression, with Breslow handling of ties. Confidence intervals were obtained from profile penalized likelihood and *p*-values from a penalized likelihood-ratio test. For the remaining conventional Cox models, *p*-values for regression coefficients were derived using the Wald test. Given the limited number of events, multivariable modelling was restricted to exploratory age- and sex-adjusted analyses. Missing data were handled using available-case analysis, with each analysis including patients with available data for the variables involved. No imputation procedures were performed. All statistical analyses were performed using R software (version 4.6.1; R Foundation for Statistical Computing, Vienna, Austria) and IBM SPSS Statistics (version 22.0; IBM Corp., Armonk, NY, USA). All statistical tests were two-sided, and a *p*-value < 0.05 was considered statistically significant.

## 3. Results

**Figure 1:**
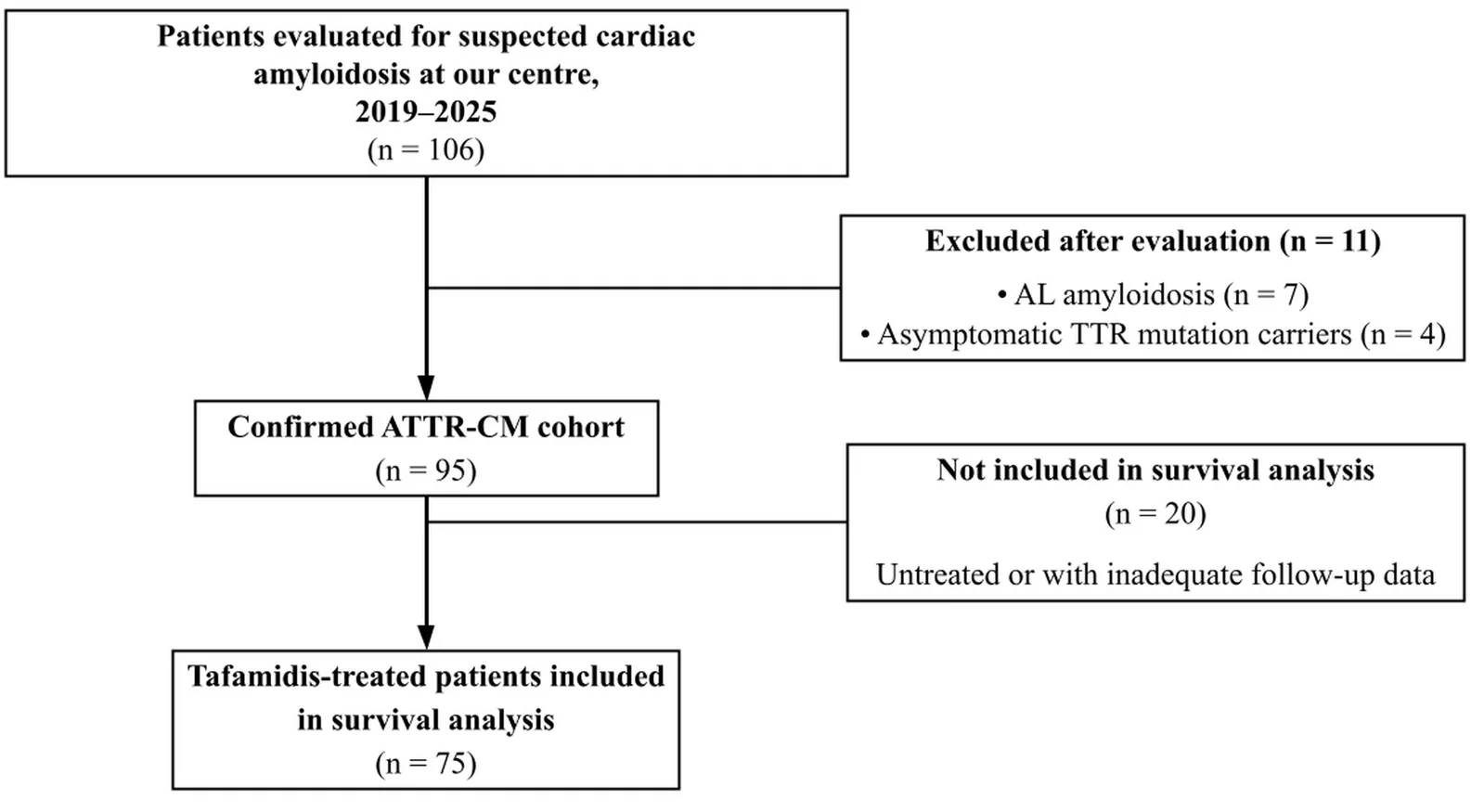
Study flowchart.

We retrospectively evaluated a cohort of 106 patients (median age 84 [IQR 76.5–88.5] years, 79% male) referred to our centre for evaluation of suspected cardiac amyloidosis between 2019–2025. After excluding 7 patients with AL amyloidosis and 4 asymptomatic TTR variant carriers, 95 patients fulfilled the diagnostic criteria for ATTR-CM. Among these, 75 patients who received tafamidis and had adequate follow-up constituted the final study cohort and were included in all subsequent analyses (Figure 1). The remaining 20 patients were excluded from the analyses, because they were untreated or had inadequate follow-up data. The majority of patients in the final cohort were male (83%) with a median age of 84 years [IQR 78.6–89.0]. Overall, 69% of patients were aged ≥80 years. ATTRv was identified in 10 patients (13%). Furthermore, 81% of patients were diagnosed from 2022 onwards.

Table 1 presents the demographic characteristics and main clinical findings of the cohort. At first evaluation, most patients had symptomatic heart failure, with 46% classified as NYHA functional classes III–IV, whereas 63% were diagnosed at NAC stage I, 16% at stage II, and 21% at stage III. At diagnosis, the median hs-cTnI level was 50 [IQR 19.4–135] pg/mL and the median NT-proBNP level was 2317 [IQR 938–4572] pg/mL. The median LVEF was 50% [IQR 40–55], the mean MWT was 15.2 ± 3.2 mm, and heart failure with preserved ejection fraction was present in 58% of patients. Of note, 5 patients had an MWT <12 mm at diagnosis. Regarding background heart failure therapy, loop diuretics were used in 64% of patients, mineralocorticoid receptor antagonists in 35%, sodium–glucose cotransporter 2 inhibitors (SGLT2is) in 29%, and sacubitril/valsartan in 9%.

Common extracardiac manifestations included bilateral carpal tunnel syndrome (33%), previous orthopedic surgery (29%), peripheral neuropathy (25%), chronic kidney disease (25%), orthostatic hypotension (17%), and spinal stenosis (15%), while severe aortic stenosis was present in 19% of the cohort. Among the 14 patients with ATTR-CM and concomitant severe aortic stenosis, 8 had undergone transcatheter or surgical aortic valve replacement before tafamidis initiation, while the rest remained untreated for severe aortic stenosis.

**Figure 2:**
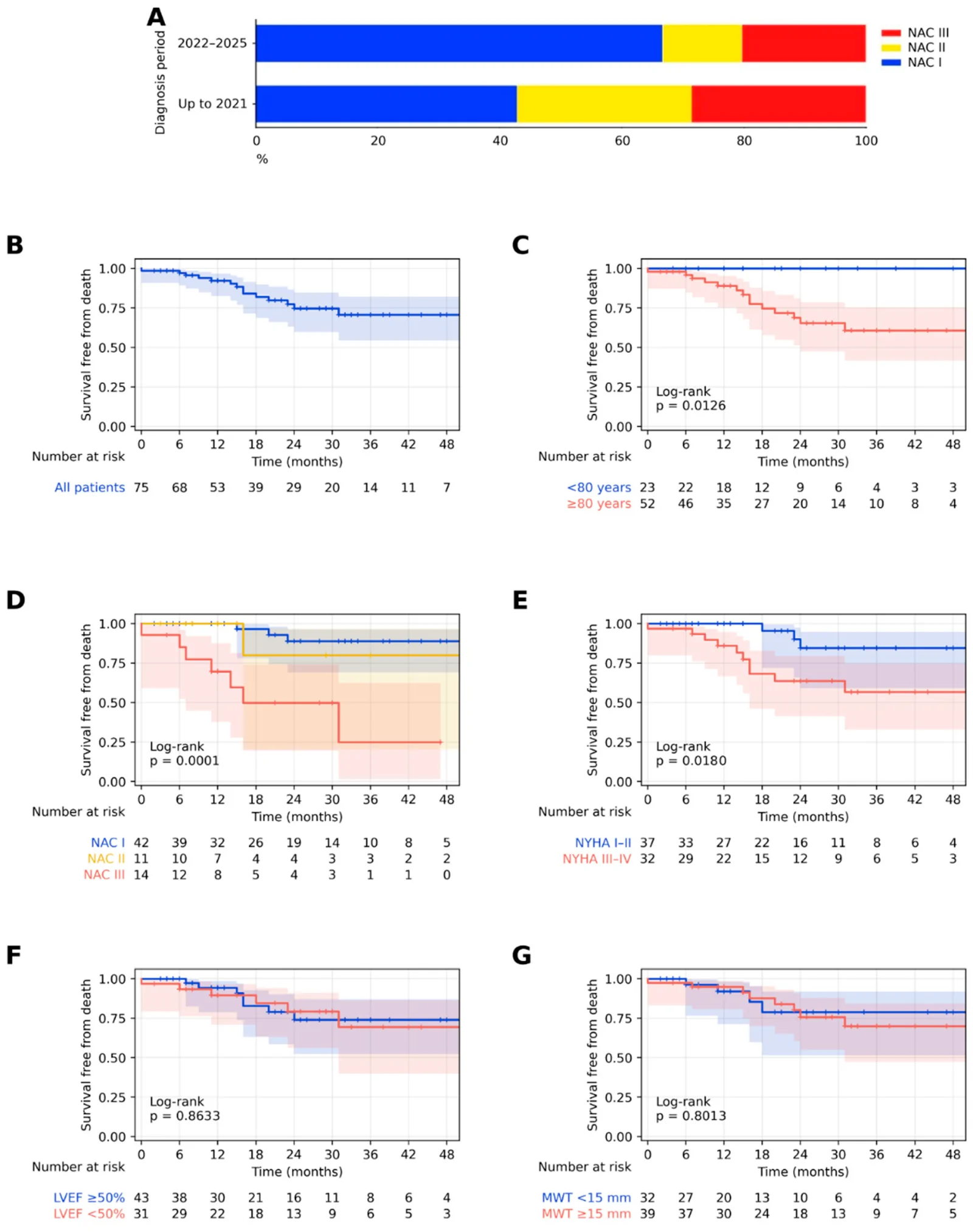
Disease stage by diagnostic period and survival analyses in patients with ATTR-CM treated with tafamidis. (**A**) Distribution of NAC disease stages at diagnosis by diagnostic period. (**B**) Overall Kaplan–Meier survival curve. (**C**) Survival analysis stratified by age (<80 vs. ≥80 years). (**D**) Survival analysis according to NAC disease stage. (**E**) Survival according to NYHA functional class (I–II vs. III–IV). (**F**) Survival according to LVEF (<50% vs. ≥50%). (**G**) Survival according to MWT (<15 mm vs. ≥15 mm). *p*-values shown in the figure are unadjusted log-rank *p*-values; the NAC comparison represents a global three-group test. Holm correction was applied across the five subgroup comparisons and is reported in the Results. Shaded areas represent 95% pointwise confidence intervals. Numbers of patients at risk are shown below each Kaplan–Meier curve. ATTR-CM: transthyretin amyloid cardiomyopathy; NAC: National Amyloidosis Centre; NYHA: New York Heart Association; LVEF: left ventricular ejection fraction; MWT: maximum wall thickness.

Among patients with available NAC staging (*n* = 67), the distribution of disease stage showed a descriptive shift over time, with a lower proportion of patients presenting with advanced NAC stage (stage III) after 2022, accompanied by a higher proportion diagnosed at NAC stage I (Figure 2A). Specifically, patients with NAC stage I increased from 43% in 2019–2021 to 68% in 2022–2025, whereas the proportion with NAC stage III decreased from 29% to 19%. However, the overall NAC stage distribution did not differ significantly between the two periods (Fisher’s exact *p* = 0.211). Ordinal logistic regression did not demonstrate a significant temporal change in NAC disease stage according to year of diagnosis (OR per calendar year 0.89, 95% CI 0.64–1.23; *p* = 0.482).

During a median follow-up period of 20 months [IQR 10.0–30.5], 14 patients (19%) died, and the estimated 30-month survival rate was 75% (95% CI 59.9–84.7%; Figure 2B). Kaplan–Meier analysis demonstrated significantly poorer survival among octogenarians compared with younger patients (log-rank *p* = 0.013), and those with NYHA classes III–IV compared with NYHA I–II (log-rank *p* = 0.018) (Figure 2C,E). Neither LVEF (<50% versus ≥50%; log-rank *p* = 0.863) nor maximal wall thickness (≥15 versus <15 mm; log-rank *p* = 0.801) was associated with all-cause mortality (Figure 2F,G). After adjustment across the five log-rank comparisons using the Holm method, only NAC stage remained statistically significant (adjusted *p* < 0.001); adjusted *p*-values were 0.050 for age, 0.054 for NYHA class, and 1.000 for both LVEF and MWT. In multivariable Cox regression, after adjustment for age and sex, NAC stage remained significantly associated with mortality (adjusted HR 3.80, 95% CI 1.82–7.92; *p* < 0.001). Detailed univariable and multivariable Cox regression results are presented in Supplementary Tables S1 and S2. 

**Figure 3:**
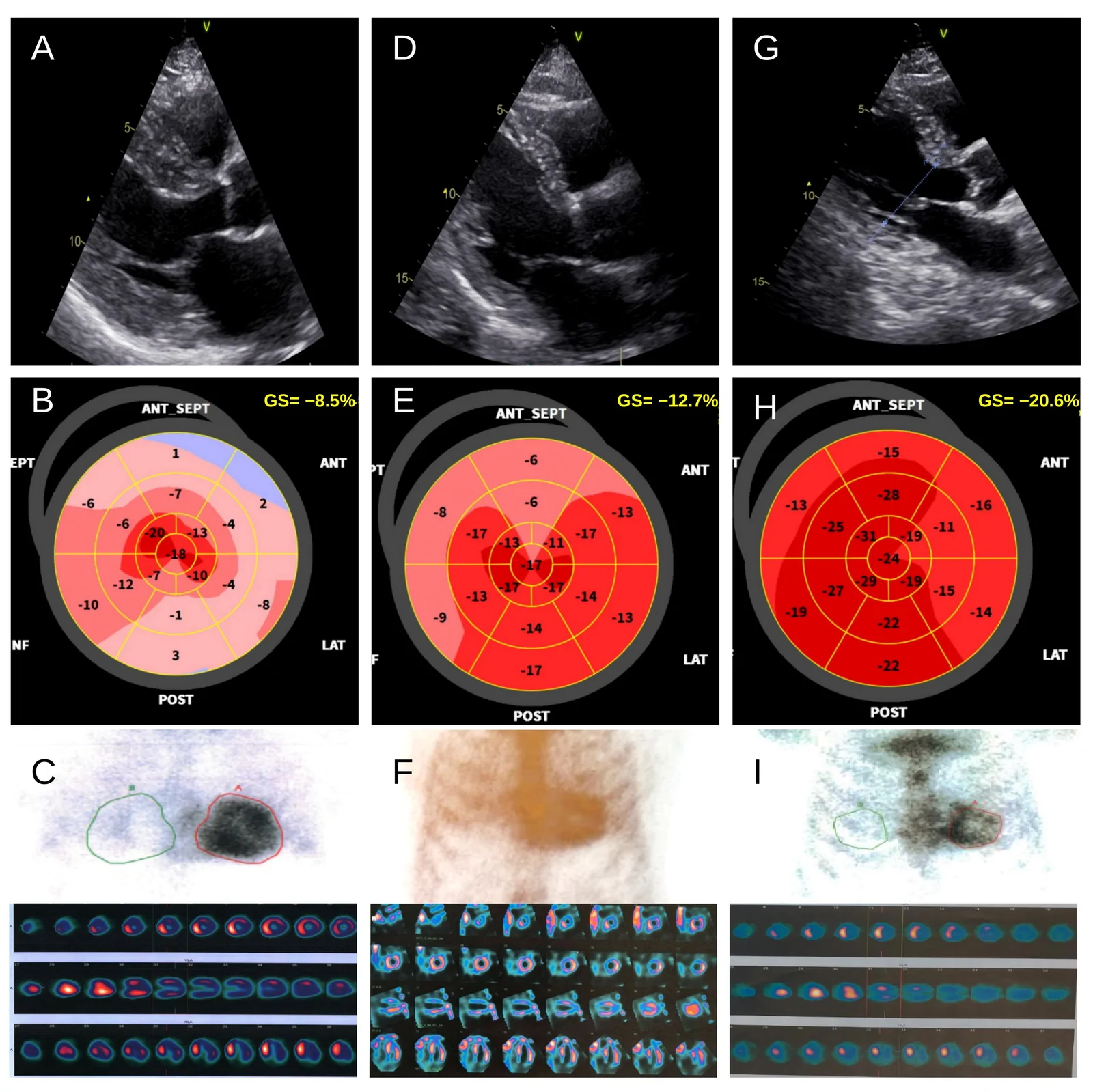
Representative multimodality imaging in three patients with ATTR-CM, illustrating the discordance between cardiac imaging phenotype and biomarker-defined disease stage. Each column represents one patient with confirmed ATTR-CM and includes parasternal long-axis echocardiography (**top**), left ventricular GLS analysis (**middle**), and 99mTc-DPD scintigraphy with corresponding SPECT (**bottom**). (**A**–**C**) Case of a 70-year-old male with marked LVH (MWT 18 mm), a characteristic apical-sparing pattern on the GLS bull’s-eye plot, and Perugini grade III myocardial tracer uptake on bone scintigraphy. Despite the advanced imaging phenotype, the patient was classified as NAC stage I (NT-proBNP 2270 pg/mL; eGFR 75 mL/min/1.73 m^2^). (**D**–**F**) Case of an 87-year-old male with mild LVH (MWT 12 mm), relatively preserved myocardial deformation with a subtle apical-sparing pattern on GLS analysis, and positive bone scintigraphy confirming ATTR-CM. The patient was classified as NAC stage I (NT-proBNP 680 pg/mL; eGFR 59 mL/min/1.73 m^2^). (**G**–**I**) Case of an 80-year-old male with no LVH (MWT 10 mm), preserved GLS without a typical apical-sparing pattern, Perugini grade III myocardial tracer uptake on bone scintigraphy, and relatively low NT-proBNP levels (400 pg/mL). The third patient with no LVH represents an illustrative atypical case of confirmed ATTR-CM. ATTR-CM: transthyretin amyloid cardiomyopathy; eGFR: estimated glomerular filtration rate; GLS: global longitudinal strain; LVH: left ventricular hypertrophy; MWT: maximum wall thickness; NAC: National Amyloidosis Centre; NT-proBNP: N-terminal pro B-type natriuretic peptide; SPECT: single-photon emission computed tomography.

Representative multimodality imaging cases are presented in Figure 3, illustrating the heterogeneity of ATTR-CM imaging phenotypes and their discordance with biomarker-defined disease stage.

## 4. Discussion

In this study, we present our centre’s experience with patients with ATTR-CM over the past six years, a period during which non-invasive diagnostic methods and access to targeted treatments became increasingly available in Greece. The estimated 30-month survival on tafamidis treatment in this cohort of ATTR-CM patients was 75%. In exploratory survival analyses, octogenarians and patients diagnosed with more advanced NAC disease stage or assessed with worse NYHA functional class exhibited poorer overall survival; however, after adjustment for multiple comparisons, only NAC stage remained significant in the log-rank analyses. Conventional echocardiographic parameters such as LVEF and maximal wall thickness were not associated with all-cause mortality.

Compared with patients receiving tafamidis in the ATTR-ACT trial, our cohort was older (median age 84 vs. 75 years) and included a lower proportion of men [12]. Median NT-proBNP levels were within a broadly similar range to those reported in ATTR-ACT (2995.9 pg/mL [IQR 1751.5–4861.5]) [12]. These differences highlight the distinct demographic and clinical profile of our real-world population compared with the selected population enrolled in the pivotal randomized trial and should be considered when interpreting the observed outcomes.

The Transthyretin Amyloidosis Outcome Survey (THAOS), the longest-running international registry of ATTR-CM patients, has provided important real-world evidence supporting the effectiveness of tafamidis therapy [16]. In THAOS, the estimated 30-month survival rate was 84% among patients treated with tafamidis compared with 70% in untreated patients. Compared with the tafamidis-treated population in THAOS, our cohort showed markers of greater disease burden at baseline, including a higher proportion of patients in NYHA functional class III (41% vs. 21.1%) and higher median NT-proBNP levels (2317 vs. 1883 pg/mL).

Given that octogenarians were underrepresented in the ATTR-ACT trial, the long-term extension (LTE) study of the ATTR-ACT trial provides important evidence supporting the efficacy of tafamidis in patients older than 80 years [17]. Furthermore, real-world data from an observational study of 710 patients with wild-type ATTR-CM indicated that tafamidis treatment significantly prolonged the survival of octogenarians compared with untreated octogenarians (3-year survival rate of 76%, *p* < 0.001) [18]. However, among octogenarians, initiation of treatment after the age of 90 years was associated with poorer outcomes, whereas NAC stage ≥3 was associated with a numerically higher but nonsignificant risk [18]. These findings should be interpreted with caution when extrapolated to our cohort, as 13% of our patients had ATTRv.

Our findings indicate that patients diagnosed at an advanced NAC disease stage exhibited a poorer prognosis, consistent with previous observations [19,20,21,22,23]. It has already been demonstrated that the NAC disease staging system has strong prognostic value in identifying tafamidis-treated patients with ATTR-CM at the highest mortality risk, yet shows limited utility in distinguishing between low- and intermediate-risk groups [23]. Functional status also remains clinically relevant in advanced ATTR-CM, although its association with mortality was not statistically significant in our cohort after correction for multiple comparisons. Since patients with NYHA class IV were excluded from the ATTR-ACT trial, data on the efficacy of tafamidis in this population have been limited [12]. The HEAR registry included octogenarians with NYHA classes III–IV, demonstrating that treated subjects with advanced heart failure had a 3-year survival rate of 61%, comparable to that of patients with NYHA class I–II symptoms. The survival rate in the HEAR registry was lower in untreated patients with severe symptoms (3-year survival rate of 35%), although this historical control group was diagnosed before tafamidis became available in France [24].

Notably, neither LVEF nor MWT was associated with all-cause mortality in our cohort. However, previous studies have reported associations for both parameters with outcomes in ATTR-CM. The LTE of the ATTR-ACT trial (median follow-up of 60–64 months) showed that continuous tafamidis treatment reduced mortality by approximately 47% compared with delayed treatment in patients with both LVEF <50% and LVEF ≥50%, underscoring the importance of early treatment initiation irrespective of LVEF [25]. In the single-centre study of Kubota et al., wall thickness analysed as a continuous variable in the Cox model was independently associated with all-cause mortality and tafamidis treatment was associated with significantly better survival in both patients with wall thickness <16 mm and those with wall thickness ≥16 mm [20].

The proportion of patients presenting with NAC stage III was numerically lower in the contemporary period, but neither the period-based comparison nor the analysis by calendar year demonstrated a statistically significant difference. Earlier recognition of ATTR-CM has been accompanied by a substantial reduction in diagnostic delay. Data from the National Amyloidosis Centre demonstrated that the median duration of symptoms before diagnosis decreased from 36 months in patients diagnosed between 2002 and 2006 to 12 months in those diagnosed between 2017 and 2021 (*p* < 0.001) [10]. The shift toward earlier diagnosis was associated with a higher proportion of patients presenting at earlier NAC stages and with milder cardiac phenotypes, including less left ventricular hypertrophy (i.e., interventricular septal thickness) and better-preserved LVEF [10,26]. The prognostic importance of early diagnosis has also been demonstrated in several studies [10,22]. In a post hoc analysis of ATTR-ACT and its LTE, patients at NAC stage I experienced earlier survival benefit than those treated at more advanced stages. Similar benefit based on NAC stage was observed in cardiovascular mortality, with survival curves separating earliest among patients with NAC stage I and progressively lower in those with NAC stages II and III. Furthermore, patients treated at NAC stages I and II experienced fewer cardiovascular hospitalizations and a slower decline in health-related quality of life, as assessed by the Kansas City Cardiomyopathy Questionnaire, compared with patients who initiated treatment at more advanced stages [22].

The landscape of ATTR-CM diagnosis and management in Greece has evolved substantially over recent years [19]. Increased awareness among cardiologists, broader implementation of non-invasive diagnostic algorithms, and improved access to bone scintigraphy and disease-modifying therapies have facilitated earlier recognition of the disease. In parallel, considerable efforts have been made to increase awareness of ATTR-CM through national research initiatives and collaborative networks. Notably, the GRECA-TAVI Registry was established to determine the prevalence of ATTR-CM among Greek and Cypriot patients undergoing transcatheter aortic valve implantation and to develop a practical risk score incorporating readily available clinical and echocardiographic variables—including age, sex, maximal wall thickness, LVEF, and the presence of an intracardiac device—to identify patients who may benefit from targeted screening for ATTR-CM [5].

Our findings regarding the association between NAC stage and survival are broadly consistent with those previously published for Greece [19]. It should be noted that the association between NYHA class and survival in our cohort did not remain statistically significant. Our treated-only cohort does not establish an increase in annual referrals or diagnoses, a significant temporal shift towards earlier-stage diagnosis, or the comparative effectiveness of tafamidis.

Despite substantial advances in the diagnosis of ATTR-CM, patients frequently experience prolonged diagnostic delays and referrals to multiple specialists before the final diagnosis is established, largely owing to the nonspecific clinical presentation and low index of suspicion among healthcare professionals [27]. Although the time to diagnosis has decreased considerably in recent years, delayed recognition remains an important clinical challenge. The DIAM-ATTR study, which evaluated the diagnostic pathway from the perspective of healthcare professionals, demonstrated that cardiologists (96%) and internists (87%) were the specialists most likely to suspect cardiac amyloidosis. However, awareness of the established ATTR-CM red flags varied substantially across medical specialties, highlighting persistent knowledge gaps even among physicians most frequently involved in the care of these patients [28]. These findings underscore the need for continued multidisciplinary educational initiatives aimed at improving recognition of ATTR-CM, facilitating earlier referral to specialized centres, and enabling timely initiation of disease-modifying therapy.

Future studies with larger multicentre cohorts and longer follow-up are required to better define long-term outcomes in patients with ATTR-CM receiving disease-modifying therapies. The establishment of a nationwide transthyretin amyloidosis registry in Greece would facilitate systematic collection of real-world data, enable benchmarking with international registries, and provide valuable insights into the effectiveness of emerging therapies, including TTR stabilizers such as acoramidis and gene-silencing agents such as vutrisiran, across different disease stages.

## 5. Strengths and Limitations

This study represents a contemporary, real-world cohort of consecutive patients with ATTR-CM evaluated at a national amyloidosis referral centre using a standardized diagnostic pathway, enhancing the consistency and reliability of diagnosis. The cohort reflects contemporary routine clinical practice at a tertiary ATTR-CM referral centre and includes a large proportion of octogenarians, who are typically underrepresented in randomized clinical trials. Furthermore, the study captures the evolving landscape of ATTR-CM diagnosis and management in the era of non-invasive imaging and disease-modifying therapy, allowing the assessment of temporal trends in disease stage at diagnosis and survival outcomes. Finally, the use of standardized disease staging and comprehensive clinical characterization facilitates comparisons with major international registries and landmark clinical trials.

This study presents several limitations. First, its retrospective, single-centre design may have introduced referral and selection bias, possibly limiting the generalizability of the findings to other populations. As a tertiary referral centre, our institution is more likely to receive patients with suspected ATTR-CM or more advanced disease. In addition, patients without available follow-up after tafamidis initiation were excluded from the survival analysis rather than censored, which may have introduced selection bias if the absence of follow-up was not random. Second, the analysis of temporal changes in disease stage was restricted to tafamidis-treated patients with available NAC staging rather than including all patients diagnosed at our centre during the study period. This may have introduced treatment-selection, survivorship, and missing-data bias, limiting the interpretation and generalizability of the observed temporal pattern. Furthermore, the modest sample size and limited number of events restricted the extent of multivariable adjustment. Additionally, the study of advanced echocardiographic markers, e.g., strain indices, remained outside the scope of this study and was not systematically assessed. Finally, although follow-up extended to a median of 20 months, longer observation is required to fully evaluate the long-term outcomes among tafamidis-treated patients, given the slowly progressive nature of ATTR-CM.

## 6. Conclusions

In this contemporary real-world cohort of Greek patients with ATTR-CM treated with tafamidis, the estimated all-cause mortality at 30 months was 25% and more advanced NAC stage was associated with poorer survival. These observations support the need to diagnose ATTR-CM as early as possible. Larger studies are needed to assess temporal changes in diagnosis and the independent associations of other clinical variables with outcomes.

## Figures and Tables

**Table 1:** Baseline characteristics of cohort patients.

Variable	ATTR-CM Patients Under Tafamidis (*n* = 75)	
Male sex (%)	83	
Age (years)	84 [78–89]	
Octogenarians (%)	69	
Time of Diagnosis (%)		
2019–2021	19	
2022–2025	81	
Genetic test performed (%)	72	
ATTR-CM subtype (%)		
ATTRwt	59	
ATTRv	13	
Unknown	28	
NYHA class baseline (*n* = 69) (%)		
I	17	
II	36	
III	41	
IV	6	
NT-proBNP (pg/mL)	2317 [938–4572]	
hs-cTnI (pg/mL)	50 [19.4–135]	
	2019–2021	2022–2025
NAC stage (*n* = 67) (%)		
I	43	68
II	29	13
III	29	19
LVEF (*n* = 74) (%)	50 [40–55]	
MWT (*n* = 71) (mm)	15.2 ± 3.2	
HFpEF (*n* = 74) (%)	58	
LVH (%)	92	
ICD (%)	8	
PPM (%)	23	
CKD (%)	25	
Severe aortic stenosis (%)	19	
CTS (%)		
Unilateral	8	
Bilateral	33	
Peripheral neuropathy (%)	25	
Orthostatic hypotension (%)	17	
Spinal stenosis (%)	15	
Biceps rupture (%)	5	
Hx of orthopedic surgeries (%)	29	
Background therapy		
Sacubitril/valsartan (%)	9	
Mineralocorticoid receptor antagonists (%)	35	
Loop diuretics (%)	64	
SGLT2i (%)	29	
Anticoagulants (%)	56	

ATTRv: hereditary ATTR; ATTRwt: wild-type ATTR; CKD: chronic kidney disease; CTS: carpal tunnel syndrome; HFpEF: heart failure with preserved ejection fraction; hs-cTnI: high-sensitivity cardiac troponin I; Hx: history; ICD: implantable cardioverter-defibrillator; LVEF: left ventricular ejection fraction; LVH: left ventricular hypertrophy; MWT: maximum wall thickness; NAC: National Amyloidosis Centre; NT-proBNP: N-terminal pro B-type natriuretic peptide; PPM: permanent pacemaker; SGLT2i: sodium–glucose cotransporter 2 inhibitors.

## Data Availability

The data presented in this study are available on request from the corresponding author, subject to approval by the relevant institutional authorities.

## Supplementary Materials

The following supporting information can be downloaded at: https://www.mdpi.com/article/10.3390/diagnostics16203373/s1. Table S1. Univariable Cox proportional hazards analysis of factors associated with all-cause mortality in tafamidis-treated patients with ATTR-CM. Table S2. Age- and sex-adjusted multivariable Cox regression analyses for all-cause mortality. File S1. STROBE Statement—checklist of items that should be included in reports of observational studies.
