# Supplementary material for: Survival Outcomes in Transthyretin Cardiac Amyloidosis in the Tafamidis Era: Real-World Evidence from Greece
Source: Diagnostics (Basel). 2026 Oct 16;16(20):3373. doi: 10.3390/diagnostics16203373 (PMC13703235; doi:10.3390/diagnostics16203373)
Supplement: Supplementary file 1 [file diagnostics-16-03373-s001.zip › STROBE-checklist 07102026.pdf]

STROBE Statement—checklist of items that should be included in reports of observational studies

	Item No.	Recommendation	Page No.	Relevant text from manuscript
**Title and abstract**	1	(*a*) Indicate the study's design with a commonly used term in the title or the abstract	2	Methods: This retrospective study included 75 patients with ATTR-CM treated with tafamidis.
		(*b*) Provide in the abstract an informative and balanced summary of what was done and what was found	2	This retrospective study included 75 patients with ATTR-CM treated with tafamidis. Baseline demographic, clinical, laboratory, and echocardiographic characteristics were assessed, and survival outcomes were subsequently evaluated
**Introduction**				
Background/rationale	2	Explain the scientific background and rationale for the investigation being reported	3	Despite being previously considered a rare entity, over the last decade ATTR-CM has gained recognition as an important cause of heart failure with preserved ejection fraction… The integration of non-invasive methods… has substantially improved disease recognition and enabled earlier diagnosis…
Objectives	3	State specific objectives, including any prespecified hypotheses	4	Therefore, we aimed to characterize temporal trends in referral patterns, disease stage at diagnosis, and clinical outcomes among patients with ATTR-CM referred to a tertiary amyloidosis centre in Greece during the tafamidis era.
**Methods**				
Study design	4	Present key elements of study design early in the paper	6	This was a retrospective, single-centre observational cohort study including consecutive patients with ATTR-CM.
Setting	5	Describe the setting, locations, and relevant dates, including periods of recruitment, exposure, follow-up, and data collection	6	...referred to the Cardiogenetics Unit of the 1st University

				Cardiology Clinic, Hippokration General Hospital, Athens, Greece, between January 2019 and March 2025.
Participants	6	(*a*) *Cohort study*—Give the eligibility criteria, and the sources and methods of selection of participants. Describe methods of follow-up<br>*Case-control study*—Give the eligibility criteria, and the sources and methods of case ascertainment and control selection. Give the rationale for the choice of cases and controls<br>*Cross-sectional study*—Give the eligibility criteria, and the sources and methods of selection of participants	6	Patients were identified through institutional registries and electronic medical records. All consecutive adult patients with a confirmed diagnosis of ATTR-CM… were eligible for inclusion.” Exclusions are also specified for AL/other amyloidosis, insufficient baseline data, missing diagnostic investigations or unavailable follow-up.
		(*b*) *Cohort study*—For matched studies, give matching criteria and number of exposed and unexposed<br>*Case-control study*—For matched studies, give matching criteria and the number of controls per case	N/A	the study was not matched.
Variables	7	Clearly define all outcomes, exposures, predictors, potential confounders, and effect modifiers. Give diagnostic criteria, if applicable	8	Baseline variables corresponded to the patient's clinical status at the time of ATTR-CM diagnosis…” followed by demographic, clinical, biomarker and echocardiographic variables; The primary outcome was all-cause mortality.
Data sources/ measurement	8*	For each variable of interest, give sources of data and details of methods of assessment (measurement). Describe comparability of assessment methods if there is more than one group	8	All patients underwent a standardized diagnostic work-up…”; laboratory assessment used measurements closest to diagnosis; hs-cTnI assay and echocardiographic measurement methods are specified.
Bias	9	Describe any efforts to address potential sources of bias	7,26	All consecutive adult patients…” and All patients underwent a standardized diagnostic work-up… The manuscript also acknowledges “referral and selection bias” due to the retrospective single-centre design.

Study size	10	Explain how the study size was arrived at	NA	No formal sample-size calculation was performed; study size was determined by the number of consecutive eligible patients during the predefined study period.

Continued on next page

Quantitative variables	11	Explain how quantitative variables were handled in the analyses. If applicable, describe which groupings were chosen and why	11-12	Continuous variables were initially evaluated for normality… Normally distributed variables are presented as mean ± SD, whereas skewed variables are summarized as median and IQR." Groupings included age <80/≥80, LVEF ≥50/<50%, and MWT ≥15/<15 mm.
Statistical methods	12	(*a*) Describe all statistical methods, including those used to control for confounding	11-12	Baseline characteristics were compared using Student's t-test or Mann–Whitney U test… Pearson's χ^2 test or Fisher's exact test… Overall survival was estimated using the Kaplan–Meier method… Cox proportional hazards regression was used…<br><br>multivariable modelling was restricted to exploratory age- and sex-adjusted analyses.
		(*b*) Describe any methods used to examine subgroups and interactions	11-12	Prespecified subgroup analyses were performed according to age (<80 versus ≥80 years), NAC stage (I, II, III), NYHA functional class (I–II versus III–IV), LVEF (≥50% versus <50%), and maximal wall thickness (≥15 mm versus <15 mm).
		(*c*) Explain how missing data were addressed	11-12	Missing data were handled using available-case analysis, with each analysis including patients with available data for the variables

				involved. No imputation procedures were performed.”
		(*d*) *Cohort study*—If applicable, explain how loss to follow-up was addressed<br>*Case-control study*—If applicable, explain how matching of cases and controls was addressed<br>*Cross-sectional study*—If applicable, describe analytical methods taking account of sampling strategy	11	Follow-up time was calculated from tafamidis initiation until death or the date of last available clinical follow-up for censored patients… Patients who remained alive… were censored at their last documented clinical contact.
		(*e*) Describe any sensitivity analyses	NA	
**Results**				
Participants	13*	(a) Report numbers of individuals at each stage of study—eg numbers potentially eligible, examined for eligibility, confirmed eligible, included in the study, completing follow-up, and analysed	14	We retrospectively evaluated a cohort of 106 patients… In total… patients with ATTR-CM… Survival analysis included 75 patients receiving tafamidis.
		(b) Give reasons for non-participation at each stage	6	Patients with evidence of… AL amyloidosis or other forms… were excluded. Patients with insufficient clinical information… missing essential diagnostic investigations, or unavailable follow-up data were also excluded…
		(c) Consider use of a flow diagram	16	Figure 1
Descriptive data	14*	(a) Give characteristics of study participants (eg demographic, clinical, social) and information on exposures and potential confounders	14	The majority of patients were male (83%) with a median age of 84 years [IQR 78–89]., Overall, 69% of the cohort were aged ≥80 years. ATTRv was identified in 10 patients (13%). Overall, 81% of patients were diagnosed from 2022 onwards.

		(b) Indicate number of participants with missing data for each variable of interest	35	Table 1 and supplementary tables
		(c) *Cohort study*—Summarise follow-up time (eg, average and total amount)	15	During a median follow-up period of 20 months [10.0–30.5]
Outcome data	15*	Cohort study—Report numbers of outcome events or summary measures over time	15	During a median follow-up of 22 months [IQR 9.0–32.5], 14 patients (18.7%) died, and the estimated 30-month survival rate was 74.7% (95% CI 59.8–84.7%) (Figure 2B). Kaplan–Meier analysis demonstrated poorer survival among patients aged ≥80 years (log-rank p = 0.013), those with NYHA class III–IV (log-rank p = 0.018), and across NAC stages I–III (global log-rank p < 0.001) (Figure 2C–E). Neither LVEF (<50% versus ≥50%; log-rank p = 0.863) nor MWT (≥15 versus <15 mm; log-rank p = 0.801) was associated with all-cause mortality (Figure 2F–G). After Holm correction, only NAC stage remained statistically significant; adjusted p-values were <0.001 for NAC, 0.051 for age, 0.054 for NYHA class, and 1.000 for LVEF and MWT.”
		Case-control study—Report numbers in each exposure category, or summary measures of exposure		
		Cross-sectional study—Report numbers of outcome events or summary measures		
Main results	16	(*a*) Give unadjusted estimates and, if applicable, confounder-adjusted estimates and their precision (eg, 95% confidence interval). Make clear which confounders were adjusted for and why they were included	Fig 2, supplementary tables	During a median follow-up period of 20 months [IQR 10.0–30.5]22 months [IQR 9.0–32.5] [9.0–32.5],

			14 patients (18.7%) died, and the estimated 30-month survival rate was 74.7% (95% CI 59.98–84.7%,) (Figure 21B). Kaplan–Meier analysis demonstrated significantly poorer survival among octogenarians compared with younger patients (log-rank *p* = 0.0126), and those with NYHA class III–IV compared with NYHA I–II (log-rank p = 0.0180) (Figure 21C-E). Neither LVEF (<50% versus ≥50%; log-rank p = 0.8633) nor maximal wall thickness (≥15 versus <15 mm; log-rank p = 0.8013) was associated with all-cause mortality (Figure 21F-G). After adjustment across the five log-rank comparisons using the Holm method, only NAC stage remained statistically significant (adjusted p = 0.000585); adjusted p-values were 0.0506 for age, 0.0539 for NYHA class, and 1.000 for both LVEF and MWT.
	(*b*) Report category boundaries when continuous variables were categorized	12	"age (<80 versus ≥80 years)… NYHA (I–II versus III–IV), LVEF (≥50% versus <50%), and maximal wall thickness (≥15 mm versus <15 mm)."
	(*c*) If relevant, consider translating estimates of relative risk into absolute risk for a meaningful time period	NA	

Continued on next page

Other analyses	17	Report other analyses done—eg analyses of subgroups and interactions, and sensitivity analyses	12, Supplementary tables	"After Holm correction across the five log-rank comparisons, only NAC stage remained statistically significant (adjusted p < 0.001). In multivariable cox-regression, after adjustment for age and sex, NAC stage remained significantly associated with mortality (adjusted HR 3.80, 95% CI 1.82–7.92; p < 0.001).
**Discussion**				
Key results	18	Summarise key results with reference to study objectives	20	In this study, we present our centre's experience with on patients with ATTR-CM over the past six years, a period during which non-invasive diagnostic methods and access to targeted treatments became increasingly widely available in Greece. The estimated 30-month survival on tafamidis treatment in this cohort of ATTR-CM patients was 74.7%. In exploratory survival analyses, octogenarians, patients diagnosed with more advanced NAC disease stage or assessed with worse NYHA functional class exhibited poorer overall survival; however, after adjustment for multiple comparisons, only NAC stage remained significant in the log-rank analyses. Conventional echocardiographic parameters such as LVEF

				and maximal wall thickness were not associated with all-cause mortality.
Limitations	19	Discuss limitations of the study, taking into account sources of potential bias or imprecision. Discuss both direction and magnitude of any potential bias	20	Second paragraph of limitations
Interpretation	20	Give a cautious overall interpretation of results considering objectives, limitations, multiplicity of analyses, results from similar studies, and other relevant evidence	20-26	Our findings indicate that patients diagnosed at an advanced NAC disease stage exhibited a poorer prognosis, consistent with previous observations…<br>The Discussion contextualizes findings against ATTR-ACT, THAOS and other cohorts.
Generalisability	21	Discuss the generalisability (external validity) of the study results	26	The cohort … includes a large proportion of octogenarians who are typically underrepresented in randomized clinical trials.” “...its retrospective, single-centre design may have introduced referral and selection bias, possibly limiting the generalizability of the findings to other populations.
**Other information**				
Funding	22	Give the source of funding and the role of the funders for the present study and, if applicable, for the original study on which the present article is based	29	The authors acknowledge funding from the Hellenic Society of Cardiology (protocol no. 20579, 24 February 2026) and from the Greek National Network of Precision Medicine in Cardiology and the Prevention of Sudden Death in the Young (project/grant no. 2018ΣΕ01300001).

*Give information separately for cases and controls in case-control studies and, if applicable, for exposed and unexposed groups in cohort and cross-sectional studies.

Note: An Explanation and Elaboration article discusses each checklist item and gives methodological background and published examples of transparent reporting. The STROBE checklist is best used in conjunction with this article (freely available on the Web sites of PLoS Medicine at http://www.plosmedicine.org/, Annals of Internal Medicine at http://www.annals.org/, and Epidemiology at http://www.epidem.com/). Information on the STROBE Initiative is available at www.strobe-statement.org.
