# Supplementary material for: Survival Outcomes in Transthyretin Cardiac Amyloidosis in the Tafamidis Era: Real-World Evidence from Greece
Source: Diagnostics (Basel). 2026 Oct 16;16(20):3373. doi: 10.3390/diagnostics16203373 (PMC13703235; doi:10.3390/diagnostics16203373)
Supplement: Supplementary file 1 [file diagnostics-16-03373-s001.zip › Supplementary tables.pdf]

Supplementary Material

Survival outcomes in transthyretin cardiac amyloidosis in the tafamidis era: real-world evidence from Greece

Freideriki Eleni Kourti,[1] Alexios S. Antonopoulos,[1] Vasileios Kytinos,[1] Theodoros Tsampras,[1] Alexandrina Kouroutzoglou,[1] Dimitrios Kyriakopoulos,[1] Maria-Eleni Koilakou,[1] Pinelopi Karampina,[2] Vasiliki Gardikioti,[1] Dimitrios Terentes-Printzios,[1] Alexandros Kasiakogias,[1] Ioannis Leontsinis,[1] Christina Chrysochoou,[1] Maria Koutelou,[3] Konstantinos Toutouzas,[1] Konstantinos Tsioufis,[1] Charalambos Vlachopoulos[1]

[1]Cardiogenetics Unit, 1st Cardiology Department, Hippokration Hospital, National and Kapodistrian University of Athens, Greece.

[2] Department of Nuclear Medicine, Kosmoiatriki, Athens, Greece

[3] Department of Nuclear Medicine, Bioiatriki, Athens, Greece

Supplementary Table S1. Univariable Cox proportional hazards analysis of factors associated with all-cause mortality in tafamidis-treated patients with ATTR-CM.

Variable	Patients with available data, n	Events, n	HR (95% CI)	P value
Age ≥80 years	75	14	**12.85 (1.72–1642.17)**	**0.006**
NYHA class III–IV	69	13	**4.18 (1.15–15.21)**	**0.030**
NAC stage (per 1-stage increase)	67	11	**3.31 (1.62–6.71)**	**0.001**
LVEF <50%	74	13	1.1 (0.37–3.28)	0.864
MWT ≥15 mm	71	12	1.17 (0.35-3.90)	0.801

CI, confidence interval; HR, hazard ratio; LVEF, left ventricular ejection fraction; MWT, maximal wall thickness; NAC, National Amyloidosis Centre; NYHA, New York Heart Association.

Supplementary Table S2. Age- and sex-adjusted multivariable Cox regression analyses for all-cause mortality.

**Variable**	**Adjusted HR (95% CI)**	**p-value**
NYHA functional class III–IV	3.00 (0.82–11.02)	0.097
NAC stages (per stage)	**3.80 (1.82-7.92)**	**<0.001**

CI, confidence interval; HR, hazard ratio; NAC, National Amyloidosis Centre; NYHA, New York Heart Association.
